# Natural Compounds as Therapeutic Agents in the Treatment Cystic Fibrosis

**DOI:** 10.4172/2157-7412.1000284

**Published:** 2016-01-30

**Authors:** Isha Dey, Kalpit Shah, Neil A Bradbury

**Affiliations:** Department of Physiology and Biophysics, Chicago Medical School, North Chicago, Illinois, USA

**Keywords:** CFTR, Natural, Genistein, Curcumin, Resveratrol

## Abstract

The recent FDA approval of two drugs to treat the basic defect in cystic fibrosis has given hope to patients and their families battling this devastating disease. Over many years, with heavy financial investment from Vertex Pharmaceuticals and the Cystic Fibrosis Foundation, pre-clinical evaluation of thousands of synthetic drugs resulted in the production of Kalydeco and Orkambi. Yet, despite the success of this endeavor, many other compounds have been proposed as therapeutic agents in the treatment of CF. Of note, several of these compounds are naturally occurring, and are present in spices from the grocery store and over the counter preparations in health food stores. In this short review, we look at three such compounds, genistein, curcumin, and resveratrol, and evaluate the scientific support for their use as therapeutic agents in the treatment of patients with CF.

## Introduction

Cystic Fibrosis (CF) is the most common lethal disease of Caucasians, and results from mutations in a cyclic AMP regulated anion channel. This ion channel, CFTR (cystic fibrosis transmembrane conductance regulator), is present in many epithelia where it regulates the transepithelial movement of salt and water [1,2]. The absence of CFTR protein and/or function in patients with CF leads to deleterious consequences in many tissues, including defective exocrine pancreatic function, intestinal blockage, and in males azoospermia. The organ most responsible for morbidity and mortality in CF is the lungs. Inappropriate salt and water transport across airway epithelia leads to the accumulation of thick sticky mucus in the lumen of the airways, which traps bacteria, causing a persistent airway infection and associated inflammation. Chronic inflammation eventually leads to tissue destruction and fibrosis, with most patients eventually succumbing to pulmonary hypertension. Following the cloning of the CFTR gene in 1989, the early hope for a therapy to treat patients with CF was founded firmly in the realm of gene therapy [3]. Indeed, several high profile gene therapy trials were initiated, yet none lived up to expectations. We now appreciate that one of the major hurdles associated with gene therapy for CF is the ability to infect efficiently epithelia cells of the airways.

Pharmacological treatments directed towards the basic defect in CF are designed to restore normal salt and water transport across affected epithelia [4]. Even moderate increases in the function of mutant CFTR are of benefit, since studies on individuals with splice variants of CFTR who exhibit only ~10% of wild-type CFTR levels appear to have normal lung function and normal life expectancy [5]. Although ~2,000 different mutations have been described in the *cftr* gene, giving rise to clinical CF, they nonetheless fall into two broad categories; those that affect protein production, and those that affect protein function [1,4]. Some mutations do in fact appear in both categories, as is the case for the most prevalent mutation, ΔF508, which constitutes about 70% of the CF chromosomes in North America [6]. Given the two broad classes of CFTR mutation, it has become apparent that two categories of drug are likely to be required to treat patients with CF, based upon their unique genetic makeup. Thus, compounds that increase the protein expression of mutant CFTR are referred to as “*correctors*”, whilst those that increase the functional activity of mutant CFTR are referred to as “*potentiators*”. In 2012, the Food and Drug Administration (FDA) of the US Government approved the first drug to treat the basic defect in CF. Marketed as Kalydeco (Vertex Pharmaceuticals), this drug was a potentiator, targeting one of the more common mutations in CF patients of Scandinavian descent, G551D. The G551D protein is characterized as a protein which inserts into the plasma membrane but has markedly reduced ion channel function. In 2015, Vertex Pharmaceuticals received FDA approval for a drug combination therapy, marketed as Orkambi. is preparation contained the same potentiator as present in Kalydeco, but also contains a “corrector” aimed at increasing protein production of mutant CFTR. This combination is primarily aimed at the common ΔF508 mutation, which displays both protein production challenges and functional problems.

A criticism of the Vertex drugs has been the pricing structure, with Orkambi and Kalydeco priced at more than $300,000 a year. Paul Quinton, a Professor of Biomedical Science at the University of California at San Diego, a pioneer in CF research and himself a CF patient has called this pricing “egregious”, a sentiment echoed by many CF clinicians, including Dr. David Orenstein, co-director of the Palumbo Cystic Fibrosis Center at Pittsburgh [7]. With this in mind, and the current trend in natural therapies, it is not surprising that many patients and their families have sought alternatives to big pharma solutions. Indeed, a growing trend amongst patients with chronic diseases, such as diabetes or CF, is the pursuit of alternative or “natural” remedies. This trend is reflected in the growing number of health food stores with advertising for a myriad of “herbal cures”. Perhaps one of the classical examples of this approach is in the treatment of chronic pain or headache, where extracts from Willow bark have proven to be beneficial. The active ingredient in such extracts is the compound salicylin, a forerunner of the modern pharmaceutical acetylsalicylic acid, or aspirin [8]. In this review, we highlight some of the proposed therapies arising from natural sources that have been proposed to be of benefit to CF patients, and evaluate their scientific merit.

### Genistein

**Figure 1:**
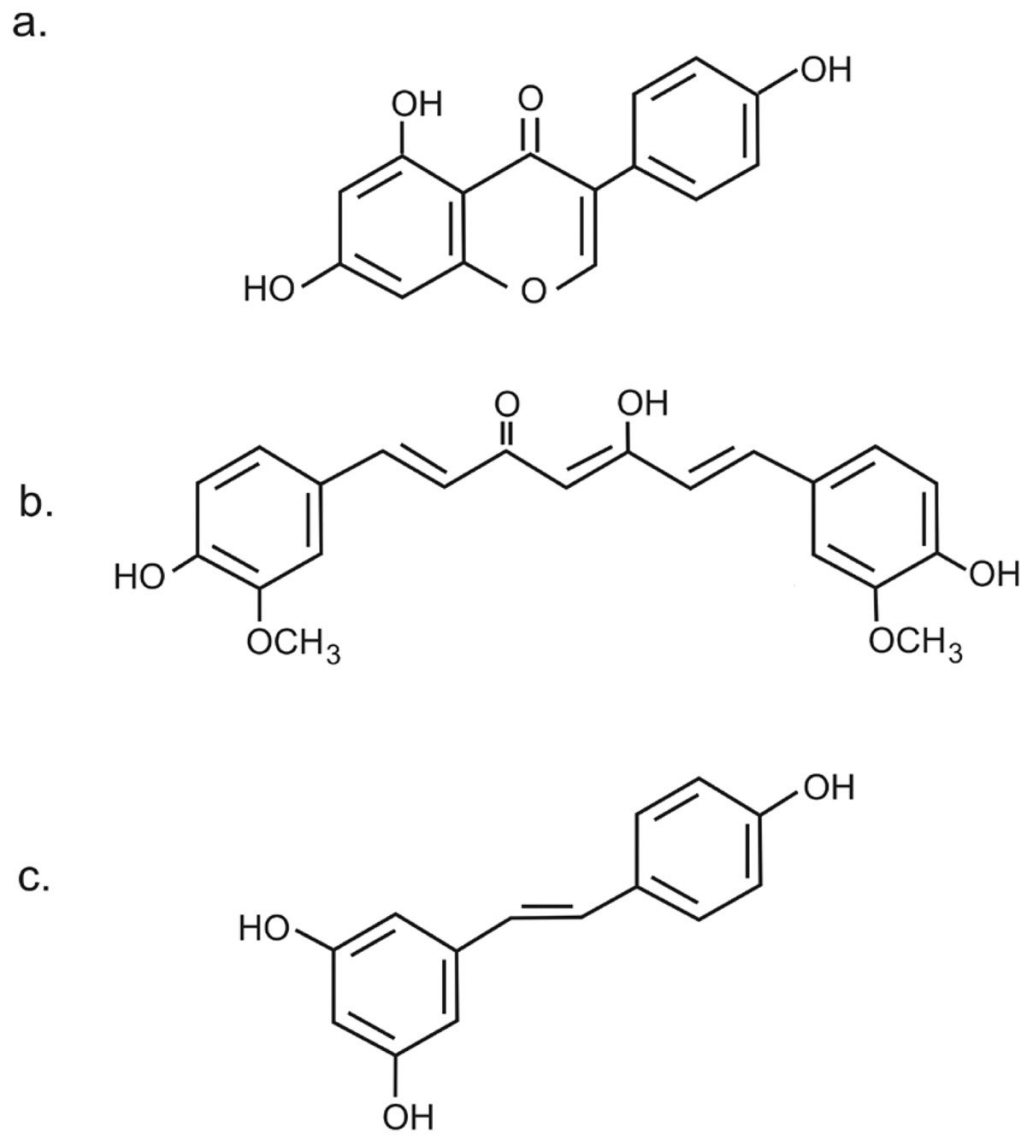
Chemical structure of naturally occurring compounds proposed for use in CF patients (a) genistein, (b) curcumin, (c) resveratrol.

One of the first compounds found to impact mutant CFTR was genistein (Figure 1a). Genistein (5,7-dihydroxy-3-(4-hydroxyphenyl)4*H*-1-benzopyran-4-one) is part of a family of compounds referred to as isoflavones, heterocyclic polyphenols found naturally in many plants [9–11]. Perhaps one of the richest sources of genistein is soya (although in soya, genestein occurs as the glycoside, genistein). Numerous health benefits have been attributed to genistein, including its actions as a phytoestrogen, an antioxidant and a tyrosine kinase inhibitor, and has been proposed to be effective in various disorders such as cancer, cardiovascular disease and menopausal problems [12,13]. Although the effects of genistein can be somewhat weak, its low toxicity has encouraged researchers to evaluate genistein as a potential therapeutic agent. The discovery that genistein could act as a “potentiator” drug raised the possibility that genistein could be used in patients with CF. Indeed, studies suggested that not only could genistein augment the ion channel activity of G551D CFTR, a gating class of mutant, but also the common ΔF508 mutation [9,14,15]. The notion that genistein might be effective against G551D CFTR is attractive, since the G551D mutation results in a protein that reaches the plasma membrane as a mature protein, but with severely impaired function [16]. Genistein appears to interact directly with CFTR to modulate its ion channel activity, and indeed is one of the more potent potentiators of ΔF508 CFTR [9,17–19]. Intriguingly, in addition to potentiating CFTR channel activity, long term treatment of cells with genistein has been shown to increase the level of protein expression for mutant CFTR as well; however 3-fold higher concentrations were found to be inhibitory [20].

Given its low toxicity, genistein appears to be a good candidate for the treatment of patients with CF. Importantly, the effective concentration for channel modulation (~2–3 μM), is within the range of achievable plasma levels of genistein (~1–2 μM) [21]. With regards to CFTR protein production, the observation that 100 μM genistein is inhibitory is somewhat irrelevant given achievable plasma concentrations. However, it also means that stimulatory concentrations of ~30 μM are also unlikely to be achievable. Although a clinical trial using a combination therapy of 4-phenylbutyrate and genistein has been planned, it was cancelled before the trial was initiated.

### Curcumin

Turmeric is a spice widely used in Asian cuisine, and has also been used for centuries as a part of the herbal therapies in Siddha medicine [22]. The principal active ingredient in turmeric is the diarylhepanoid curcumin (Figure 1b), a compound which give turmeric its characteristic yellow colouring. Indeed, curcumin is widely available as a nutritional supplement, reported as efficacious as having anti-inflammatory, anti-tumour and antioxidant effects [23–25]. *In vitro*, curcumin has been shown to inhibit a number of enzymes, including HDAC1,3,8, cyclooxygenase, and importantly for ΔF508 CFTR, the sarcoplasmic/endoplasmic reticulum calcium pump (SERCA) [26–31]. Inhibition of SERCA by curcumin presumably blocks ATP-dependent uptake of calcium into the endoplasmic reticulum, thus interfering with calcium-dependent processes within the ER, including a number of calcium-dependent chaperones. In fact, earlier studies had shown that the SERCA inhibitor thapsigargin could facilitate ER exit of ΔF508 CFTR, with subsequent appearance of the mutant protein in the plasma membrane, where it could be available for activation [32]. Exposure of baby hamster kidney cells, transfected with human ΔF508 CFTR, to curcumin was reported to improve the processing of ΔF508 CFTR allowing to exit the ER and insert into the plasma membrane [33]. Moreover, administration of oral curcumin to ΔF508 CF mice, resulted in sufficient correction of ΔF508 CFTR trafficking, that normalized nasal potential-difference measurements could be attained; including a reduction in the level of epithelial sodium transport (a process which is thought to be a significant contributor to CF lung pathology) [33,34]. Intestinal obstruction, a hallmark of CF disease in many mouse models (presumably due to congestion of the gut by reduced water transport into the gut lumen) and a major cause of death in CF mice, was also corrected in CF mice exposed to curcumin. The intriguing consequence of these studies was the notion that a single, simple, agent was capable of correcting ΔF508 CFTR in a clinically beneficial manner. Given that curcumin is found in various foods, and is sold as a herbal remedy, the idea that clinical trials based on a compound with hundreds of years of biosafety data could be undertaken is very appealing.

Given the exciting results initially obtained, other groups attempted to replicate the studies, with varying degrees of success, with one group unable to duplicate the nasal potential-difference measurements [35]. This group was also unable to show the corrective effects of curcumin *in vitro*, whereas other correctors, including genistein, were effective [35]. Why different groups get contrary observations is still not clear, but obviously must be addressed prior to any clinical trials with curcumin. One difference is the genetic background of the ΔF508 mice used in the studies. The positive studies by Egan et al. were performed on a mixed background (129/sv and C57BL/6); whereas the negative studies of Song et al. were on a CD-1 background [34,35]. Precisely how this divergent background might contribute to the varying results is still, however, not clear. Another confounding issue is the achievable plasma concentrations for curcumin, and important issue in human trials. In Phase 1 clinical trials, dietary curcumin has been shown to exhibit very poor bioavailability, which coupled with rapid metabolism and excretion means a very low serum concentration. Song et al. reported a plasma concentration of 60 nM for curcumin, whereas the known IC50 for curcumin on SERCA is orders of magnitude higher at 5–15 μM [35–39]. This, of course, does not rule out the possibility that curcumin may be doing something to ΔF508 CFTR other than impacting its interaction with calcium-dependent chaperones, but clearly raises the issue as to whether SERCA could be a target for curcumin and physiologically relevant plasma concentrations.

### Resveratrol

Resveratrol (Figure 1c) has recently received attention as the primary ingredient contributing to the health benefits associated with red wine. Resveratrol (3,4′,5-trihydroxystilbene) is a naturally occurring polyphenolic compound found in vegetables and fruits, and abundant in grapes and peanuts [40]. Similarly to curcumin, resveratrol is widely available in health food stores, and is reported to be effective due to its anti-mutagenic, anti-inflammatory, anti-oxidant and chemo-protective properties [41,42]. The mechanism(s) by which resveratrol achieves the effects are not well documented, however it is known that resveratrol can increase cellular cAMP levels through direct activation of adenylate cyclase and by inhibiting cAMP phosphodiesterases [43,44]. Several reports, in cell lines, primary mouse tissues, and in mouse nasal potential difference studies, have shown that resveratrol can increase the ability of ΔF508 CFTR to exit the ER and traffic to the cell surface and be functional [45–48]. Such studies reported an increase in band b to C conversion for ΔF508 CFTR, and salutary effects including increased airway fluid secretion and mucocilliary clearance. One interesting observation was that resveratrol appeared to increase the activity of ENaC, enhancing absorptive sodium transport, potentially further exacerbating the enhanced sodium hyper-absorption seen in CF airways [47,49]. In the hands of other researchers, resveratrol was able to increase wt CFTR expression, but was unable to increase ΔF508 CFTR expression in expression systems [50]. Using primary human airway epithelial cells from patients homozygous for the ΔF508 CFTR mutation, our studies were unable to demonstrate any benefit from resveratrol exposure, even though known “correctors” were effective [50]. Moreover we were also unable to see any effects on amiloride sensitive sodium currents, suggesting that ENaC was not a target for resveratrol. Interestingly, resveratrol by itself could stimulate chloride secretion across a human colonic monolayer, a stimulation that was markedly enhanced by the addition of a small amount of forskolin. Such observations are at least consistent with the hypothesis that resveratrol can enhance CFTR activity by acting as a phosphodiesterase inhibitor [50,51]. It is possible that resveratrol works directly on CFTR by acting as a potentiator, indeed, resveratrol has been reported to increase the open probability (Po) of murine CFTR, although it should be noted that murine CFTR has different electrophysiological properties than human CFTR [47,52]. Intriguingly, although monomeric resveratrol can increase CFTR activity, oligomeric resveratrol is a CFTR inhibitor [53].

What accounts for the differences in these studies using resveratrol? At present it is not entirely clear, however there are certainly differences in cell models used. Another issue is the concentration of resveratrol used in the studies. The majority of studies seeing efficaciousness of resveratrol do so at concentrations >50 μM. Indeed the studies of Jai et al. also see an effect of resveratrol on wt CFTR at concentrations above 50 μM [50]. However, as with curcumin, the issue of effective *in vitro* concentration versus achievable plasma concentration is an issue that has to be addressed. Although beneficial effects for resveratrol are reported at concentrations about 50 μM, the maximal achievable plasma concentration is ~2 μM, even with high dose oral administration [42,54,55]. When physiologically relevant levels of resveratrol were applied to primary human CF tissue, no beneficial effects on chloride transport were observed [50]. Thus, although resveratrol may be useful in cell models, its current use in humans seems premature.

## Discussion

What should be the response of CF patients and their families to these natural compounds discussed above? Should patients be placed on a steady diet of curries and red wine? It is an unfortunate truth that many preparations of natural remedies are not standardized, nor do they always contain the level of active ingredient that they are purported to contain. Furthermore such remedies are not subject to regulatory oversight, as are drugs from pharmaceutical companies. However, it is also true that while the current pricing for FDA approved CF drugs from Vertex Pharmaceuticals is ~$300,000 per year, supplements such as genistein, curcumin and resveratrol can be obtained for a few hundred dollars per year. Certainly for curcumin and resveratrol, the achievable plasma concentrations are significantly lower that can be obtained through oral supplementation. Whether chemical modifications to increase absorption and/or bioavailability can be achieved remains unclear. Similarly, what structural changes could be made to improve the potency of these compounds remains to be determined. If they were to be made, such modifications would likely have to come from academia. The reluctance from pharmaceutical companies to embrace many potential natural therapies stems, in part, from difficulties in biosynthesis and subsequent purification. Such problems would only be compounded when medicinal chemistry to modify the structures is initiated, if indeed it is technically possible to design synthetic pathways to generate such compounds in a cost-effective manner.

Given the wide availability of the naturally occurring compounds discussed, it is not surprising that CF patients are willing to test such compounds on themselves. At best it is likely that such self-administration is without effect. What interactions between these compounds and FDA approved drugs might occur remains unknown. Indeed, while compounds such as genistein, curcumin and resveratrol may not present any clinical complications, the uncertainty with what other components may be in the over-the-counter preparations is cause for concern. Does this mean that all natural compounds should be dismissed? This idea is probably too severe. What is important is that the exact mechanistic actions by which such compounds impinge on mutant CFTR to cause it to traffic and/or function better be understood. Such knowledge has the potential to impact on a rational design of synthetic drugs for CFTR, such that ultimately a safe, effective and inexpensive drug is available to treat patients with CF.
